# Effectiveness of a Digital Screening and Navigation Model in Addressing Unmet Social Needs among Parents and Caregivers in Priority Population Groups: A Randomised Controlled Trial

**DOI:** 10.5334/ijic.9228

**Published:** 2026-07-02

**Authors:** Teresa Winata, James John, Si Wang, Melissa Smead, Weng Tong Wu, Jane Kohlhoff, Virginia Schmied, Bin Jalaludin, Kenny Lawson, Siaw-Teng Liaw, Raghu Lingam, Andrew Page, Christa Lam-Cassettari, Katherine Boydell, Ping-I Lin, Ilan Katz, Ann Dadich, Shanti Raman, Rebekah Grace, Aunty Kerrie Doyle, Tom McClean, Blaise Di Mento, John Preddy, Susan Woolfenden, Valsamma Eapen

**Affiliations:** 1 Faculty of Medicine and Health, Discipline of Psychiatry and Mental Health, University of New South Wales, Sydney, NSW, Australia; 2 Ingham Institute for Applied Medical Research, Liverpool, NSW, Australia; 3 National Disability Insurance Scheme Quality and Safeguards Commission, Parramatta, NSW, Australia; 4 Academic Unit of Infant, Child and Adolescent Psychiatry, South Western Sydney Local Health District, Liverpool, NSW, Australia; 5 Research and Evaluation Group, The Salvation Army, Sydney, NSW, Australia; 6 Murrumbidgee Local Health District, Wagga Wagga, NSW, Australia; 7 Karitane, Carramar, NSW, Australia; 8 School of Nursing and Midwifery, Western Sydney University, Parramatta, NSW, Australia; 9 South Western Sydney Local Health District, Liverpool, NSW, Australia; 10 Faculty of Medicine and Health, School of Public Health and Community Medicine, University of New South Wales, Sydney, NSW, Australia; 11 School of Business, Parramatta, Western Sydney University, NSW, Australia; 12 WHO Collaborating Centre for eHealth, University of New South Wales, Sydney, NSW, Australia; 13 Faculty of Medicine, Population Child Health Research Group, School of Women’s and Children’s Health, University of New South Wales, Sydney, NSW, Australia; 14 School of Medicine, Western Sydney University, Sydney, NSW, Australia; 15 Black Dog Institute, Sydney, NSW, Australia; 16 Department of Psychiatry and Behavioral Neuroscience, School of Medicine, Saint Louis University, Missouri, USA; 17 Faculty of Arts, Social Policy Research Centre, Design, and Architecture, University of New South Wales, Sydney, NSW, Australia; 18 Transforming early Education and Child Health Research Centre, Western Sydney University, Penrith, NSW, Australia; 19 Uniting NSW.ACT, Sydney, NSW, Australia; 20 Rural Clinical School, School of Clinical Medicine, University of New South Wales, Wagga Wagga, NSW, Australia; 21 Faculty of Medicine and Health, Sydney Medical School, University of Sydney, Sydney, NSW, Australia; 22 Sydney Local Health District, Sydney, NSW, Australia

**Keywords:** digital health, child development, psychosocial care, unmet needs, service navigation

## Abstract

**Introduction:**

Children and families from priority populations (e.g. culturally and linguistically diverse and regional/rural communities) often experience significant psychosocial challenges and barriers to accessing health and social care. These inequities were further exacerbated during the COVID-19 pandemic. Integrating social care with health services has been proposed as an approach to improve service access and address unmet needs.

**Methods:**

A two-site parallel randomised controlled trial was conducted in Australia between August 2021 and 2023. Parents/carers of children from priority populations were recruited through Child and Family Health services (n = 288) and randomised to the Watch Me Grow–Electronic (WMG-E) intervention (n = 145) or care as usual (n = 143). The WMG-E program comprised digital developmental screening and community navigation to relevant health and social services via a service navigator. The primary outcome was change in unmet social needs measured using the WE CARE instrument. Intention-to-treat generalised linear mixed-effects models adjusted for child developmental concerns, parental mental health, and sociodemographic factors.

**Results:**

Unmet social needs decreased over time across both groups (β = –0.23, SE = 0.11, p = 0.036). At 12 months, the intervention group showed greater reductions in unmet needs than the control group (β = –0.35, SE = 0.17, p = 0.046), although the time × group interaction was not significant.

**Discussion:**

The WMG-E platform demonstrated feasibility as a digital developmental screening and navigation tool supporting families from priority populations.

**Conclusion:**

WMG-E shows promise in improving access to health and social care and reducing unmet social needs; however, further research is needed to assess sustained impacts across diverse settings.

**Trial registration:**

The study (Protocol No. 1.0, Version 3.1) was registered with ANZCTR (registration number: ACTRN12621000766819) on July 21^st^ 2021, and the trial results are being reported according to recommendations in the CONSORT Statement.

**Summary Box:**

1. **What is already known on this topic**
  - Significant inequities exist in the access to healthcare for priority population families with preschool children and this results in children missing opportunities for early identification and intervention for health and developmental problems.
  - While addressing heath care inequities is critical for providing children a healthy start to life, there are no effective and systematic ways to reach such families as they do not engage with services early, and often experience significant barriers due to social care needs.
  - Addressing health inequities by tackling social determinants of health alongside healthcare is critical to supporting families in priority population groups, aligning with the Sustainable Development Goals (SDGs) of the World Health Organization (WHO).
2. **What this study adds**
  - This study is one of the first Australian randomised controlled trials evaluating the effectiveness of a digital developmental screening program coupled with service navigation for improving unmet social care needs in multicultural and rural/regional communities.
  - Significant reductions in unmet need scores were observed over time in both intervention and control groups, with a greater reduction in the intervention group, though no significant interaction between time and group was found.
  - The Watch Me Grow Electronic (WMG-E) platform was shown to be a feasible and effective tool for developmental screening and navigation to address unmet social needs in diverse, priority population families.

## Introduction

Priority populations are defined as specific groups within society who experience social disadvantage, poorer overall health, and inequities—such as avoidable and unfair differences in health status [1]. Certain groups, including individuals from low socio-economic backgrounds [2], culturally and linguistically diverse (CALD) communities [3], and those living in rural or regional areas [4], often face inequities in access to services and are at greater risk of morbidity and mortality compared to the broader population.

Evidence indicates that these populations encounter a range of financial, structural, and cultural or linguistic barriers that contribute to poorer health and wellbeing outcomes [5,6]. Increasingly, governments and health service providers are recognising these inequities and are implementing strategies to deliver more integrated and equitable care, particularly for marginalised and minority groups [7]. Efforts to address health inequities are also aligned with the Sustainable Development Goals (SDGs) of the World Health Organization (WHO) [8].

People who are born overseas and speak a language other than English are often referred to as culturally and linguistically diverse (CALD) communities in Australia [9]. Several studies have shown that people from these communities typically experience multiple social disadvantages and face challenges in health care access and in meeting their social care needs [10,11]. CALD communities, especially new migrants, refugees and asylum seekers, often face challenges in their new environment, particularly due to language barriers and consequent unemployment [12,13,14]. Barriers to help-seeking, including stigma associated with receiving help is also prevalent in various cultures and communities where seeking help for mental health and social care concerns may stem from fear of judgment, shame, or perceived weakness [15,16]. Additionally, cultural beliefs about health and mental health issues may impact help-seeking behaviours. Furthermore, the recent COVID-19 deaths in Australia also revealed a three-fold higher mortality rate among CALD communities compared to the general population [17] alongside higher rates of COVID-19 hospitalisations due to multiple co-morbid conditions and late presentations [18]. The high rates of COVID-19 related deaths and avoidable hospitalisations among these populations suggest the need to address the disadvantage and associated inequity in access to health and social care services. One way of addressing health inequity is to support priority families with preschool children early in life as it offers the best chance of optimising their life-long health and social trajectory outcomes.

Residents of Australia’s rural and remote communities also experience poorer health outcomes compared to their metropolitan counterparts [4]. Rates of potentially preventable diseases and hospitalisations increase significantly with geographical remoteness [19]. Mortality rates, possibly the best indicator of the health of the population, are significantly higher in very remote areas compared to major cities [20]. These outcomes reflect both the high proportion of socioeconomically disadvantaged and Indigenous residents with high disease burden, and inequitable access to health and social care services among those living in rural and remote communities [4,21]. Australia is not alone in striving for greater equity of access to social care and health services for those residing outside its urban areas. Both Canada and the United States, with their vast landscapes and scattered rural and remote communities, share similar health disparities that are linked to social determinants and poor access to services [22,23].

Unmet social needs, such as food and financial insecurity or housing concerns have the potential to adversely impact health, social functioning, and societal participation [24]. Health inequities experienced during early childhood have lasting impacts on future health and well-being [25]. Despite the growing knowledge about the health inequities and adverse health impact of unmet social needs, particularly for families from priority populations, there is limited understanding of how, when, and where intervention (timeliness and appropriateness) may positively impact, including the frequency and types of needs, and socio-demographic associations. International paediatric groups including the UK National Health Service, American Academy of Paediatrics (AAP), the Royal College of Paediatrics and Child Health (RCPCH), and the Royal Australasian College of Physicians (RACP) have all recognised unmet social needs as drivers of child health inequity, and an important focus for intervention by child health professionals [26,27].

A “virtual care approach” [28] was widely adopted during the COVID-19 pandemic which was largely driven by the need to ensure provision of acute care services to as many as possible during lockdown without the need for face-to-face contact [29,30]. However, as the lockdown period extended, this focus also shifted to include prevention and health promotion services such as developmental checks. In this regard, our previous research had shown an inverse care law with those from priority population such as CALD and rural/regional backgrounds, with children having higher risk for developmental problems were experiencing greater health care inequity [31,32,33] and not accessing developmental surveillance programs [34]. As approximately 3.6 billion people use social media, the use of web-based applications has become ubiquitous, including among those from priority populations [35]. Therefore, by providing a web-based application which is accessible, affordable, and easy to use, we expected to improve the access and uptake of child developmental screening in some of the most vulnerable populations in Australia. In this regard, the Watch Me Grow–Electronic (WMG-E) Platform is an innovative web-based screening and ongoing monitoring program that provides unique opportunities to reach vulnerable families at their homes and communities and thereby enable an integrated approach to supporting child developmental, parental mental health and family social care needs [36,37]. WMG-E uses opportunistic contacts that the family has with trusted service providers, such as childhood immunisation visits or routine service contact with General Practitioners (GPs), playgroups, social care agencies, government, and non-governmental organisations (NGOs) in order to empower parents to engage with child developmental checks. The program aims to identify child development needs while also ascertaining and de-stigmatising mental health and psychosocial screening, and provide urgent targeted interventions, particularly using digital technology [36,37]. Originally developed as a child developmental screening and monitoring tool, in the context of COVID-19 pandemic and consequent child and family service closure, this was expanded to include parental mental health and family psychosocial screening [37] with the goal of linking families to services [34] in a timely manner. Further, the use of opportunistic contacts with trusted service providers helped to optimise parental engagement with developmental health services particularly for those from priority populations.

An important first step was to develop standardised approaches that reach all families, including those who may not otherwise engage with prevention and health promotion services, such as child health and developmental checks [38,39]. A second priority was to identify and systematically address unmet social needs to support optimal health and functional outcomes for children and their families, particularly those from priority populations. Accordingly, this study aimed to determine whether digital care navigation using the WMG-E platform is effective in improving outcomes—by addressing unmet social needs—for priority population groups, compared with those receiving Care as Usual (CaU).

## Methods

### Study design and sites

This study employed a multisite, parallel group, randomised controlled trial (RCT) conducted in South Western Sydney Local Health District (SWSLHD) and Murrumbidgee Local Health District (MLHD) representing predominantly CALD/low-income and rural/regional communities respectively. Outreach to various stakeholders across the sites began in mid-2021 with members of the WMG-E team (e.g. chief investigators, service navigators, project manager, health services managers, and other researchers) being involved in building collaborative inter-sectorial relationships across the two geographical regions.

### Participants – eligibility criteria, recruitment, and consent

Parents/caregivers with a child aged six months to three years old attending Child and Family Health Services (CFHS), refugee health services, supported playgroups, parenting groups, Non-Government Organisations (NGOs) providing child and family health services, GP practices, specialist clinics, etc. were approached to participate in this study. Information about the study and consent procedures were presented using the WMG-E platform via a QR code and informed consent was obtained. Recruitment period took place during the COVID-19 pandemic, which was August 2021 to May 2022.

### Randomisation and blinding

Upon completion of the consent forms, participants were randomised by the WMG-E platform to either the intervention (WMG-E) or Care as Usual (CaU) group. Computerised randomisation software was used to create a randomisation table via REDCap (a secure web application for building and managing online surveys and databases). Participants were assigned randomly to each arm ensuring a 50:50 ratio.

### Intervention: Watch Me Grow-Electronic program—a digital screening and service navigation model covering the first 2000 days (from pregnancy to start of school)

Participants who were allocated to the intervention group received the WMG-E digital platform providing screening and service navigation support. Once engaged, the WMG-E platform sends automated reminders to take the developmental checks again at the next age when the child is recommended to complete the checks from birth to the start of school (i.e. 6, 12, 18, 24, 36, 48 and 60 months) as per the state government Personal Health Record program.

Risks relating to child development, parental mental health and family psychosocial needs were screened and determined via the following measures within the WMG-E platform (See Data Collection section). The full study protocol of this program has been published elsewhere^37^. Based on the scores on these measures, a risk level was determined as none, low, medium, or high, as outlined in **Supplementary Table 1**. A service navigator (SN) was allocated to each family in the intervention group. An example of the WMG-E platform results page listing the relevant resources is presented in **Supplementary Figure 1**.

The WMG-E program supported children and families via service navigators at the respective two sites by coordinating care for child development, parental mental health, and psychosocial needs as per WMG-E screening results **(Supplementary Figure 2)**. Three SNs (two bilingual SNs from a CALD background at SWSLHD site and a local SN from the rural/remote area in the MLHD site) were employed as part of this program who had extensive experience in public health (psychologist, midwifery, and/or nursing). Based on the needs identified using the screening measures, the SN used their knowledge of the healthcare system to link families with relevant supports in the community. SNs offered continuity of care before, during, and after service link up ensuring ‘warm handover’ by guiding families to appropriate services ensuring that the families have engaged with the relevant services and that their needs are being met. The role of the SN was to build relationships, identify unmet needs and support families while they learnt to self-navigate the health system and other support services. To improve coordination of child and family health care, the SNs worked with different stakeholders. While some families’ needs were met by commonly available services, others needed creative solutions to overcome service access barriers, by locating and sourcing appropriate resources or providing adaptations. The SNs helped patients “navigate” the maze of clinical, administrative and support services. While they do not provide any clinical responsibilities or services per se, the SN’s role was to support families to access timely intervention and supports for any identified child development, parental mental health or social care needs including housing, financial, education, employment, and food insecurity.

A WMG-E standard procedure manual was developed to guide its operation of service navigation and collection of participant information. The document, alongside other relevant templates, served as a way to systematise the collection of participant data for research purposes and to standardise the clinical operations of the program, while also protecting the individualised nature of care.

### Care as Usual (CaU)

Participants allocated to the control condition of CaU group received routine standard care, which included only the WMG-E digital developmental screening without the service navigation or continuity of care support. Families were provided general resources and anticipatory guidance in the form of health literacy material (using webpage links) and relevant local service provider contact information. **Supplementary Figure 1** shows samples of WMG-E platform results page listing the relevant resources.

### Data collection

All eligible participants completed a short demographic form and baseline screening measures. Given the large, anticipated number of multicultural participants in the South-Western Sydney region, the weblink was also made available in languages other than English, namely Vietnamese, Arabic, and simplified Chinese (Mandarin). Based on parental responses to the screening questions, they were then provided with their results and relevant resources. Respondents were allowed to complete the survey once, and only one respondent was permitted per household. This study involved collection of data at three timepoints: baseline (T0), six-months post-baseline (T1), and 12-months post-baseline (T2).

### Measures

While the primary outcome of the original program trial was changes in the child developmental concerns, this specific study focuses on changes in *unmet social needs* measured using the standardised WE CARE tool – while adjusting for key covariates including child developmental concerns, parental mental health, and other sociodemographic as confounding factors. These outcomes are described below.

### Primary outcome in this study: unmet social needs

The WE CARE survey is a brief, social determinants of health screening tool used to identify the level of unmet social need [40]. It focuses on four key areas: economic stability, education, neighbourhood and physical environment, and food security. The survey comprises six binary (yes/no) responses related to family psychosocial needs, covering areas such as childcare, employment, homelessness, food security, education, and utilities. Additional questions were included for participants who respond affirmatively to any of the initial six items, prompting participants to indicate, on a three-point scale (yes, no, or maybe later), whether they require further assistance in addressing their unmet psychosocial needs. Unmet needs were coded as none (WE CARE score = 0), low level (WE CARE score = 1–2), and high level (WE CARE score ≥3). The measure has good psychometric properties, including excellent test-retest reliability (r. = 0.92) [40].

### Kessler Psychological Distress Scale (K10) assessment

The K10 assessment is a widely used self-report questionnaire designed to measure psychological distress in individuals [41]. It comprises 10 items with ratings on a five-point scale assessing various aspects of emotional well-being and mental health over the past four weeks.

### Learn the Signs Act Early (LTSAE) assessment

The LTSAE [42] assessment, developed by the Centres for Disease Control and Prevention (CDC), aims to empower parents and caregivers in monitoring a child’s development from two months to five years old. The assessment includes several domains of social and emotional, language/communication, cognitive, and movement/physical development.

### Sociodemographic information

The sociodemographic questionnaire collected at baseline included: child-specific characteristics including age (in months), gender (female, male), and country of birth (English, non-English speaking countries). Parent-specific characteristics included mother’s and father’s age (in years), highest level of education (Postgraduate/graduate diploma/ Bachelors; Advance diploma/Certificate 3/4; Year 12 and/or below), CALD status (yes, no); and country of birth (English, non-English speaking countries). Family characteristics included marital status (Married, others), the main language spoken in the home (English, non-English), current service use (no, yes), and study site (South West Sydney and Murrumbidgee).

## Sample size calculation

A sample of 244 families was estimated to yield a power of over 99%, ensuring the ability to detect a significant increase in service utilisation from 30% to 60% in the intervention group (based on pilot data) with statistical significance set at 5%. Factoring in non- and partial adherence, along with a 5% allowance for sample dropout, the adjusted sample size was recalculated to be 300 (with 150 in each group).

## Data analysis

Descriptive analyses such as independent samples t-test and chi-square tests were used to determine any significant differences in demographic, clinical characteristics, and the number and type of unmet needs between the intervention and CaU groups at baseline. Homogeneity of variances was assessed by the Levene’s test for equality of variances. The primary analysis included an intention-to-treat (ITT) generalised linear mixed effects model to examine the effects of the WMG-E intervention on WE CARE scores. Linear mixed-effect models can effectively address correlations within subjects and manage imbalanced data, rendering them highly suitable for the analysis of randomised controlled trial (RCT) data with missing values [43]. Participants were specified as random effects, and time was specified as a fixed effect in mixed models. Intervention group, and a time by intervention group interaction were also assessed separately for the outcome variable whilst adjusting for key covariates. A series of pairwise contrasts were conducted to compare group differences at different follow-up periods. All analyses were undertaken in Statistical Package for the Social Sciences (SPSS) version 28 (IBM SPSS, IBM Corp., Armonk, NY, USA) and R studio version.

## Results

### Participant characteristics

**Figure 1:**
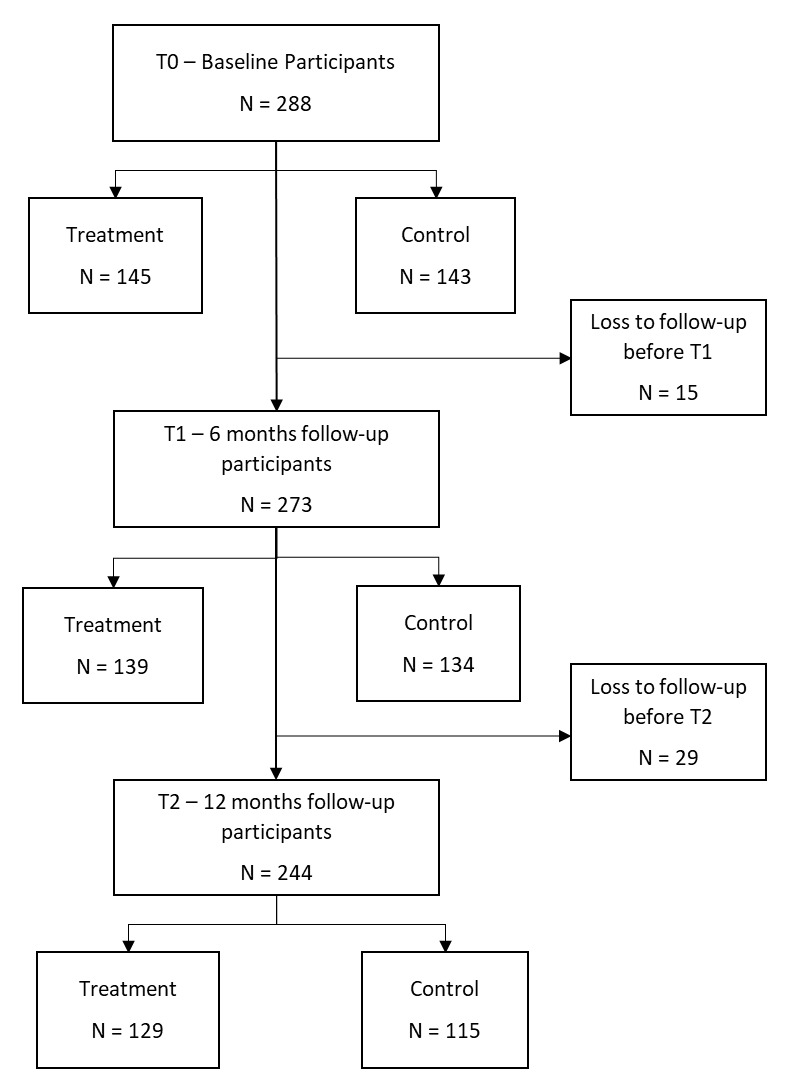
CONSORT Study Flow Diagram.

The demographic and clinical characteristics of the participants by treatment groups at baseline are shown in Table 1. The CONSORT flowchart showing randomisation of participants along with follow-up at different time points is summarised in Figure 1. Of the total sample of 288 participants, 145 participants were randomised to the intervention group whereas 143 participants were allocated to the control group. The mean age of the children in the sample was 19 months with equal male to female distribution. The sociodemographic and clinical indicators were similar across both groups with no statistically significant differences.

**Table 1:** Descriptive characteristics of participants (total sample and by treatment group).


VARIABLES	TOTAL SAMPLE (N = 288) n (%) OR M(SD)	CONTROL GROUP(N = 143) n (%) OR M (SD)	INTERVENTION GROUP(N = 145) n (%) OR M (SD)

**Sociodemographic and sociocultural factors**			

Child’s age in months, mean (SD)	18.84 (9.42)	19.07 (9.29)	18.61 (9.57)

Child’s gender			

*Female*	145 (50.3)	74 (51.7)	71 (49.0)

*Male*	143 (49.7)	69 (48.3)	74 (51.0)

Parent/Caregiver’s age in years, mean (SD)	31.23 (6.50)	30.92 (7.19)	31.54 (5.75)

Parent/Caregiver’s country of birth			

English-speaking country	208 (82.5)	102 (82.9)	106 (82.2)

Non-English-speaking country	44 (17.5)	21 (17.1)	23 (17.8)

Parent/Caregiver’s preferred language			

English	254 (88.5)	127 (89.4)	127 (87.6)

Non-English	33 (11.5)	15 (10.6)	18 (12.4)

CALD status^#^			

*No*	206 (81.7)	101 (82.1)	105 (81.4)

*Yes*	46 (18.3)	22 (17.9)	24 (18.6)

Parent/Caregiver’s education level			

*Postgraduate/Graduate diploma/Bachelor’s degree*	148 (51.4)	79 (55.2)	69 (47.6)

*Advanced Diploma/ Certificate III/IV*	78 (27.1)	40 (28.0)	38 (26.2)

*Up to Year 12*	62 (21.5)	24 (16.8)	38 (26.2)

Marital status			

*Married*	178 (62.0)	93 (65.5)	85 (58.6)

*Other (De facto/Single/Divorced)*	109 (38.0)	49 (34.5)	60 (41.4)

Site			

*Liverpool, Fairfield, Bankstown*	61 (21.2)	30 (21.0)	31 (21.4)

*Murrumbidgee*	227 (78.8)	113 (79.0)	114 (78.6)

Current service use			

*No*	211 (73.3)	108 (76.1)	103 (72.5)

*Yes*	73 (25.3)	34 (23.9)	39 (27.5)

**Clinical indicators**			

K10 scores at baseline	18.47 (6.78)	18.52 (6.82)	18.41 (6.76)

No of LTSAE concerns at baseline	0.27 (0.66)	0.29 (0.70)	0.26 (0.62)


^#^CALD status – parent’s country of birth and/or preferred language; M – Mean; SD – Standard deviation; LTSAE – Learn The Signs, Act Early; K10 – Kessler’s psychological distress scale.

### Comparison of unmet needs

The prevalence of unmet needs by intervention and control groups across the time-points are shown in Table 1. Children’s average age was approximately 19 months, and caregivers were around 31 years old, with comparable gender distribution and education levels. Most caregivers were from English-speaking countries (82.5%), preferred English (88.5%), and had balanced representation of marital status and cultural diversity across the groups.

**Figure 2:**
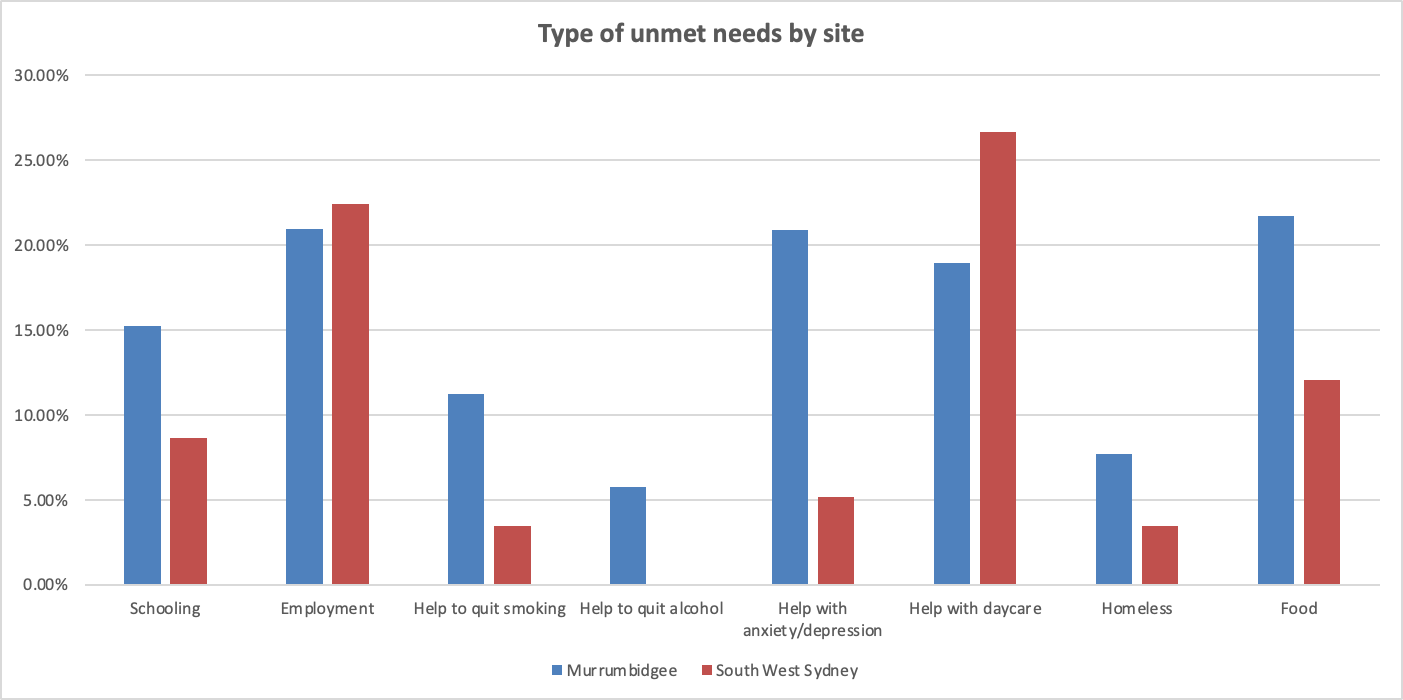
Type of unmet needs at baseline by site.

The differences in the number and type of unmet social needs by treatment group over time is presented in Table 2. There were no statistically significant differences in the mean WE CARE scores between the treatment group the control group at any timepoint. The main concerns reported by participants from MLHD included help with food, mental health, and employment whereas participants in SWSLHD reported help with day care, employment, and food (shown in Figure 2).

**Table 2:** Differences in the number and type of unmet social needs by treatment group over time.


WE CARE SCORES	INTERVENTION GROUPMEAN (SD)	CONTROL GROUPMEAN (SD)	MEAN DIFFERENCE (95% CI)	t STATISTIC	p-VALUE

***Total scores***					

T0	1.10 (1.50)	1.31 (1.48)	–0.22 (–0.56, 0.13)	–1.242	0.215

T1	1.14 (1.77)	1.19 (1.47)	–0.04 (–0.43, 0.35)	–0.211	0.833

T2	0.95 (1.57)	1.03 (1.29)	–0.08 (–0.45, 0.28)	–0.434	0.664

***Type of concerns***					

Schooling at T0	0.23 (0.64)	0.24 (0.63)	–0.01 (–0.20, 0.18)	–0.113	0.910

Schooling at T1	0.16 (0.53)	0.16 (0.49)	0.01 (–0.15, 0.16)	0.064	0.949

Schooling at T2	0.14 (0.49)	0.11 (0.43)	0.03 (–0.12, 0.19)	0.403	0.688

Employment at T0	0.43 (0.79)	0.32 (0.69)	0.11 (–0.11, 0.33)	0.966	0.335

Employment at T1	0.36 (0.75)	0.36 (0.72)	–0.01 (–0.23, 0.22)	–0.030	0.976

Employment at T2	0.28 (0.65)	0.25 (0.63)	0.03 (–0.18, 0.25)	0.350	0.727

Help to quit smoking at T0	0.17 (0.55)	0.12 (0.41)	0.05 (–0.09, 0.20)	0.729	0.467

Help to quit smoking at T1	0.15 (0.52)	0.16 (0.54)	–0.01 (–0.18, 0.15)	–0.217	0.829

Help to quit smoking at T2	0.24 (0.63)	0.07 (0.35)	0.16 (–0.01, 0.33)	1.937	0.055

Help to quit alcohol at T0	0.04 (0.24)	0.08 (0.34)	–0.04 (–0.13, 0.06)	–0.814	0.417

Help to quit alcohol at T1	0.08 (0.35)	0.08 (0.35)	–0.01 (–012, 0.11)	–0.035	0.972

Help to quit alcohol at T2	0.08 (0.36)	0.09 (0.42)	–0.01 (–0.15, 0.12)	–0.171	0.864

Help with anxiety/depression at T0	0.25 (0.61)	0.24 (0.59)	0.01 (–0.17, 0.19)	0.161	0.436

Help with anxiety/depression at T1	0.16 (0.53)	0.30 (0.66)	–0.14 (–0.32, 0.05)	–1.440	0.152

Help with anxiety/depression at T2	0.22 (0.58)	0.15 (0.50)	0.07 (–0.11, 0.26)	0.817	0.415

Help with day care at T0	0.33 (0.64)	0.28 (0.60)	0.05 (–0.12, 0.22)	0.603	0.547

Help with day care at T1	0.24 (0.56)	0.22 (0.52)	0.02 (–0.13, 0.18)	0.328	0.743

Help with day care at T2	0.12 (0.38)	0.12 (0.38)	0.01 (–0.11, 0.12)	0.056	0.955

Homeless at T0	0.07 (0.26)	0.06 (0.29)	0.01 (–0.08, 0.09)	0.207	0.836

Homeless at T1	0.06 (0.24)	0.13 (0.41)	–0.07 (–0.18, 0.04)	–1.268	0.207

Homeless at T2	0.07 (0.25)	0.06 (0.30)	0.01 (–0.09, 0.10)	0.086	0.932

Food at T0	0.23 (0.49)	0.22 (0.51)	0.01 (–0.14, 0.16)	0.116	0.908

Food at T1	0.22 (0.52)	0.30 (0.55)	–0.08 (–0.25, 0.08)	–0.972	0.333

Food at T2	0.15 (0.39)	0.25 (0.55)	–0.09 (–0.26, 0.06)	–1.245	0.215


T0 – Baseline, T1 – six-months post-baseline, T2 – 12-months post-baseline.

### Changes in the level of unmet needs

**Figure 3:**
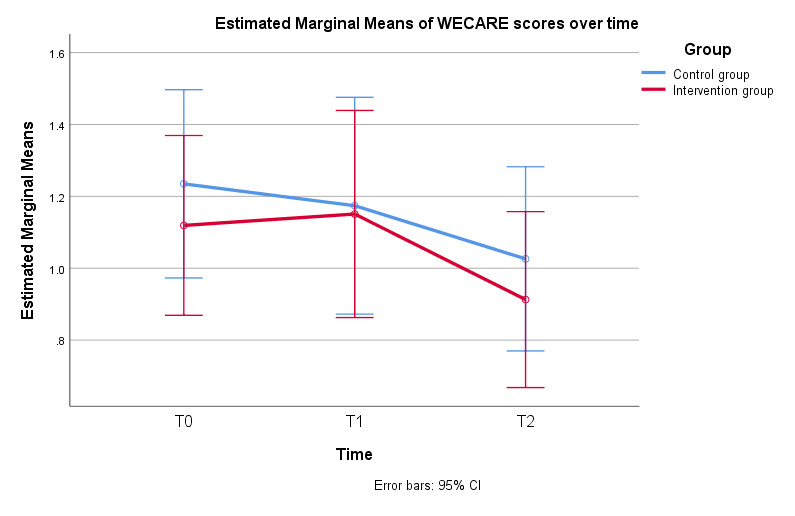
Changes in WECARE scores by treatment and control group.

Findings of the ITT generalised linear mixed-effects model (Table 3) showed significant differences in the WE CARE scores over time (within group changes) with the unmet need score reducing in both the intervention and control group over time (β = –0.23, SE = 0.11, p = –0.036). In addition, there were significant differences between intervention and control group (between group changes) (β = –0.35, SE = 0.17, p = –0.046) with a lower unmet need score in the intervention group compared to the control group. However, there was no significant interaction effect between group and time (Figure 3).

**Table 3:** Results of the Generalised Linear Mixed Model Analyses of Time-by-Group Effects as Three-Timepoint Analysis.


OUTCOME – WE CARE SCORE	β	SE	p-VALUE

Intercept			

Treatment (Ref = Control)	–0.35	0.17	**0.046**

Time (Ref = T0)			

T1	–0.11	0.11	0.305

T2	–0.23	0.11	**0.036**

Time (Ref = T0) × Treatment (Ref = Control)			

Time (T1) × Treatment (Intervention)	0.17	0.15	0.257

Time (T2) × Treatment (Intervention)	0.05	0.15	0.743


Subjects as random effects with time and group as fixed effects and adjusted for covariates: Child’s age, gender, parent’s age, education level, marital status, CALD status and site. Ref: Reference category. T0 – Baseline, T1 – six-months post-baseline, T2 – 12-months post-baseline.

Consistent with the linear mixed effects model, pairwise comparisons showed significant changes in the estimated marginal means of the WE CARE scores within groups over time (Time 1 and Time 3 as well as Time 2 and Time 3) as well as between the treatment groups (**Supplementary Table 2**).

## Discussion

### Summary of findings and comparison with existing literature

Findings of the generalised linear mixed model showed significant improvement in the psychosocial needs (WE CARE scores) over time (within group), as well as between intervention and control groups (between group), with the intervention group experiencing higher improvement in the scores. That is, the unmet social care needs of those families who were randomised in the intervention group (i.e. subjected to the full WMG-E digital screening platform and service navigation model) reduced over time. Furthermore, our clinical indicators were shown to be significantly associated with unmet psychosocial needs. It was found that higher mental health issues (K10 scores) in parents and higher number of developmental concerns (LTSAE) in their children were significantly associated with higher level of unmet needs. Compared to those with higher education level, those with lower education level had higher odds of unmet needs. Similarly, in terms of a household stability, compared to parents who were married, those parents and caregivers who were in the other category (single/de facto/divorced) were significantly associated with higher odds of unmet needs. Regarding child’s gender, those with male children were associated with higher odds of unmet needs. In contrast to families from a multicultural area of SWS, parents and/or caregivers from the Murrumbidgee region were more likely to report less unmet needs than their counterparts even though there were no significant association at the univariate level.

Our study findings are consistent with previous research which suggest that delivery of curated, individualised resources and support for child and family care can positively affect family circumstances and child and caregivers’ health [44,45,46]. Indeed, our study results displayed similar patterns to Garg et al.’s cluster RCT [47] around assessing the impacts of WE CARE screening and referral systems on families’ receipt of community-based resources for unmet basic needs in multiple urban community health settings. For instance, consistent with our study, those mothers in the WE CARE (intervention) group had lower odds of being in a homeless shelter at 12-months follow up [47]. Additionally, a previous qualitative study has informed the benefits of implementing the WE CARE screening and navigation model through the lens of clinicians and staff, and how they integrated it into the context of a busy, urban, community health centre setting led by a community paediatric unit [48]. The authors reported that this improved their ability to better serve their patients and their communities.

### Implications for health policy and practice and future research

Large-scale trials integrating early psychosocial screening within Child and Family Health settings are essential to identify and address risks for children and families early. Effective policy must support integration between health and social care systems to improve access and establish a holistic, equitable service network [49,50,51]. Programs such as our RISE framework (Responsiveness, Integration, Sustainability, Equity), emphasise prioritising families from high-need groups, suggesting that this model when embedded within routine healthcare can improve access and sustainability [52,53].

Investments in early childhood services, particularly in co-located care hubs that provide both health and social supports, can address equity gaps by reaching families via leveraging opportunistic contact points—such as health, early childhood education, and social services [54,55,56,57]. Programs like WMG-E could utilise these touchpoints to engage families in developmental screening, and thereby addressing access barriers through integrated digital navigation tools that help overcome challenges like transportation and clinic accessibility and availability of appointments. Leveraging such opportunistic contacts offers significant reach thereby addressing the current inequity in service access. WMG-E by identifying children and families with needs early can help them to be prioritised for further assessments thereby increasing service efficiency and allocation of resources for those who need it the most.

Further, investments in social care referral services are vital to ensure appropriate capacity and timely response for priority populations. Effective screening and referral systems should incorporate coordination across care levels, clear communication protocols, trained personnel, efficient data systems, and regular performance monitoring to avoid delays or unmet needs due to waitlists [58,59]. Such an approach of health care with wrap around social care systems in primary, community, secondary and tertiary care settings will ensure that families receive timely, appropriate care.

The WMG-E program is an example of how digital navigation and opportunistic contacts (e.g. routine developmental checks) can enhance integrated child development, parental mental health and psychosocial monitoring, particularly for priority populations who might otherwise miss these services. By automating follow-ups and offering continuity in care, this digital model provides a scalable, effective solution for equitable access to health and social services, which proved especially beneficial during the COVID-19 pandemic.

### Strengths and limitations of this study

Our study has several notable strengths including the RCT design and adequate sample size with high retention rate at the 12-month follow-up in both groups (85%) in a population that is usually very difficult to reach, demonstrating priority populations can be engaged in research studies. Another strength is the well balanced and comparable characteristics between the two groups at baseline. Furthermore, the use of standardised, reliable and valid measures during each data collection time-points adds credibility to our results, reflecting the practical application of early child developmental screening programs like the WMG-E platform.

However, this study has several limitations. Despite randomisation, baseline differences were observed, with higher parental education and a greater proportion of married caregivers in the control group. These factors may influence families’ ability to independently access services and may have reduced the observable impact of the navigation component. Attrition may also have affected results, as families with greater needs are often more likely to withdraw from longitudinal studies. The WE CARE survey relied on self-reported unmet social needs, which may be subject to reporting bias. The tool was developed in the USA and implemented with minimal modification to preserve fidelity to the validated instrument. Some items, such as those referring to a “high school diploma,” may be less applicable in the Australian context, where educational pathways differ. In our sample, a high proportion of caregivers reported education beyond secondary school, which may have reduced the sensitivity of this item. Future research should consider contextual adaptation and validation of the WE CARE tool for Australian settings.

The relatively low rates of unmet needs may reflect recruitment through Child and Family Health services, which already function as early access points for support, and the fact that some structural barriers (e.g., transport access and service waitlists) are not captured in the WE CARE instrument. In addition, the study was conducted during the COVID-19 pandemic, when many community and support services were closed or operating with limited capacity. This likely reduced the level of support that could be provided to families in the intervention group and may have diminished the observable differences between the intervention and control groups. Finally, while follow-up occurred at six and twelve months, longer-term follow-up is needed to assess sustained impacts on family wellbeing and child developmental outcomes. Future work should prioritise the development and validation of contextually adapted social needs screening tools alongside integrated digital navigation models to strengthen early identification and support for families experiencing social and developmental vulnerabilities.

## Conclusion

The WMG-E program was found to be feasible and effective as an online developmental screening and navigation tool for addressing unmet social needs and improving service accessibility for diverse and priority populations, including CALD and socioeconomically disadvantaged families. The study revealed significant reductions in unmet needs scores in both intervention and control groups over time, with the intervention group achieved slightly lower scores at the 12-month follow-up, though baseline differences may have contributed. While there was no evidence of a differential effect in unmet needs changes between groups, these results highlight the value of the WMG-E approach in facilitating developmental monitoring and providing holistic care during critical periods. Future research should aim to optimise and scale the intervention, explore its benefits in different populations, and investigate long-term outcomes to fully understand its impact on family experiences.

## Transparency Declaration

The lead author affirms that this manuscript provides an honest, accurate, and transparent account of the study being reported. All significant aspects of the study have been included, and no important elements have been omitted. Any discrepancies from the study as originally planned and, if applicable, registered have been clearly explained.

## Additional File

The additional file for this article can be found as follows:

10.5334/ijic.9228.s1 Supplementary Online Content. Supplementary eTables S1 to S3 and eFigures S1 to S4.

## Data Availability

Data available: Yes Data types: De-identified patient data How to access data: The research data supporting this study was collected by our research team and can be made accessible upon request. It is governed by the NHMRC Statement and authorised by the South Western Sydney Local Health District Human Research Ethics Committee. Researchers interested in accessing these data should contact the corresponding author, Professor Valsamma Eapen. When available: With publication
