# Supplementary material for: Effectiveness of a Digital Screening and Navigation Model in Addressing Unmet Social Needs among Parents and Caregivers in Priority Population Groups: A Randomised Controlled Trial
Source: Int J Integr Care. 2026 Jul 2;26(3):2. doi: 10.5334/ijic.9228 (PMC13331036; doi:10.5334/ijic.9228)
Supplement: Supplementary Online Content. — Supplementary eTables S1 to S3 and eFigures S1 to S4. [file ijic-26-3-9228-s1.pdf]

SUPPLEMENTARY ONLINE CONTENT

Supplementary eTable 1. WMG-E participant risk level assessment and referral pathway

**Scores**<br>**Risk Level**	**K10**	**WE CARE**	**LTSAE**	**Program referral pathway**
**None = 0**	<20	0	0	No referral
**Low = 1**	20 - 24	1	1	Refer – acceptable
**Medium = 2**	25 - 29	2	2	Refer – moderate<br>NOTE: Those who only scored high-risk level in ONE screening measure = 'medium' risk-level (overall)
**High = 3**	= or >30	= or > 3	= or > 3	Refer – acute crisis<br>NOTE: Those who are classified as HIGH RISK or acute crisis will need to achieve high risk/ scores/concerns in 2-3 of the screening measures.

Supplementary eFigure 1. Samples of WMG-E platform results page listing the relevant resources

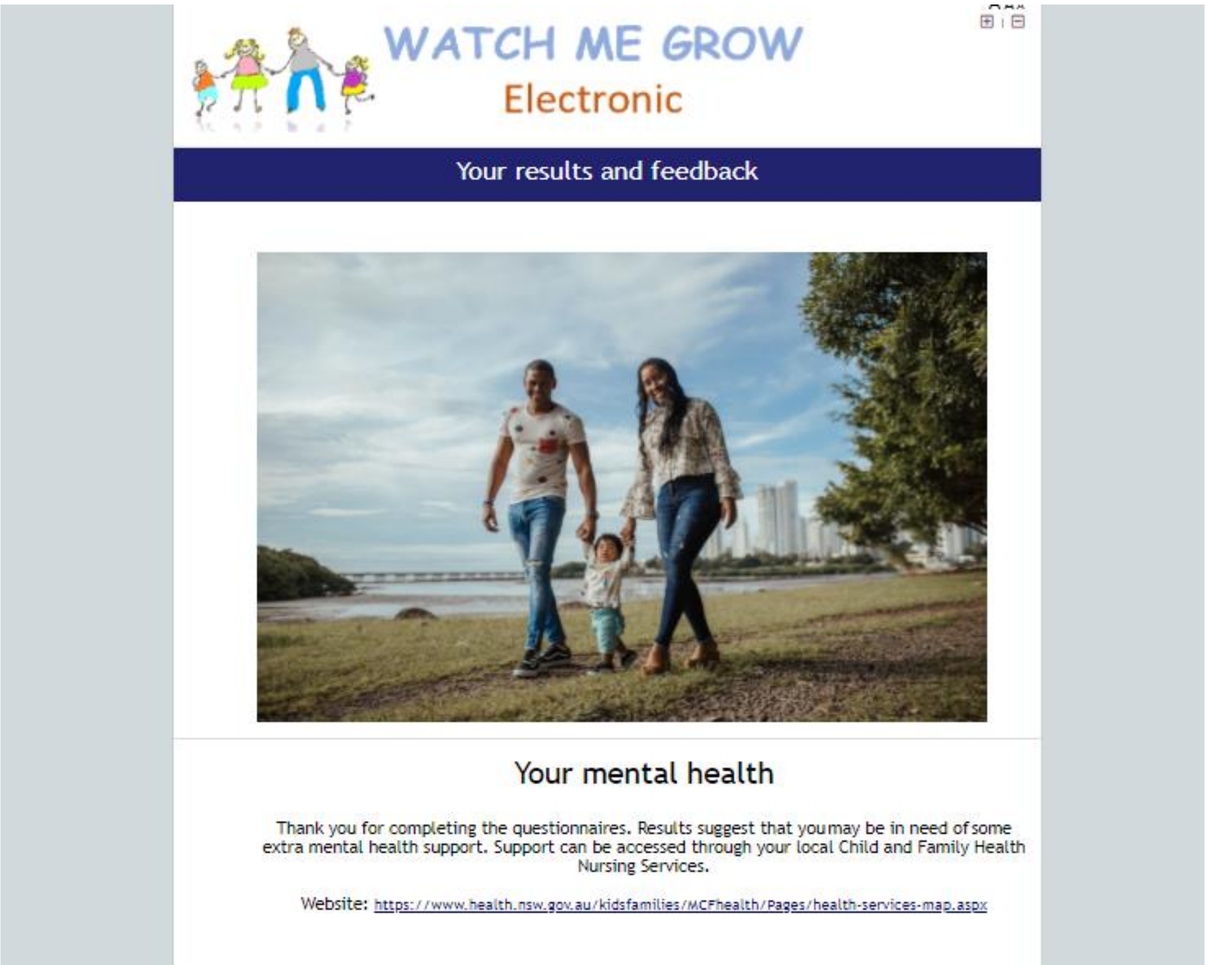

Your wellbeing needs

Thank you for completing the questionnaires. You have selected 'YES' to the following questions:

Q1: Would you like help to attend TAFE?

See the online resource about TAFE support:

- TAFE https://www.tafensw.edu.au/

Q2: Would you like help with finding employment?

See the following online resources about EMPLOYMENT support:

- Job Active https://jobactive.gov.au/
- Job Jumpstart https://www.jobjumpstart.gov.au/
- Services Australia https://www.servicesaustralia.gov.au/individuals/job-seekers

Q3a: Do you smoke cigarettes?

Q3b: Would you like help to quit smoking?

Your Child's Development

Your answers indicate that your child could benefit from a more detailed assessment. Please discuss these results with your health professional.

All children grow and develop at their own pace. Please use the following link to access information on what is expected for your child's age:

Raising Children Network

The following items came up as indicating concern about your child:

6 Months: Does your child respond to sounds around them?

6 Months: Has your child lost skills they once had?

Please have a look at the following information, describing what is expected for your child's age

Social and Emotional

- May be afraid of strangers
- May be clingy with familiar adults
- Has favorite toys

Language/Communication

Supplementary eFigure 2. The Watch Me Grow-Electronic (WMG-E) service navigation program framework.

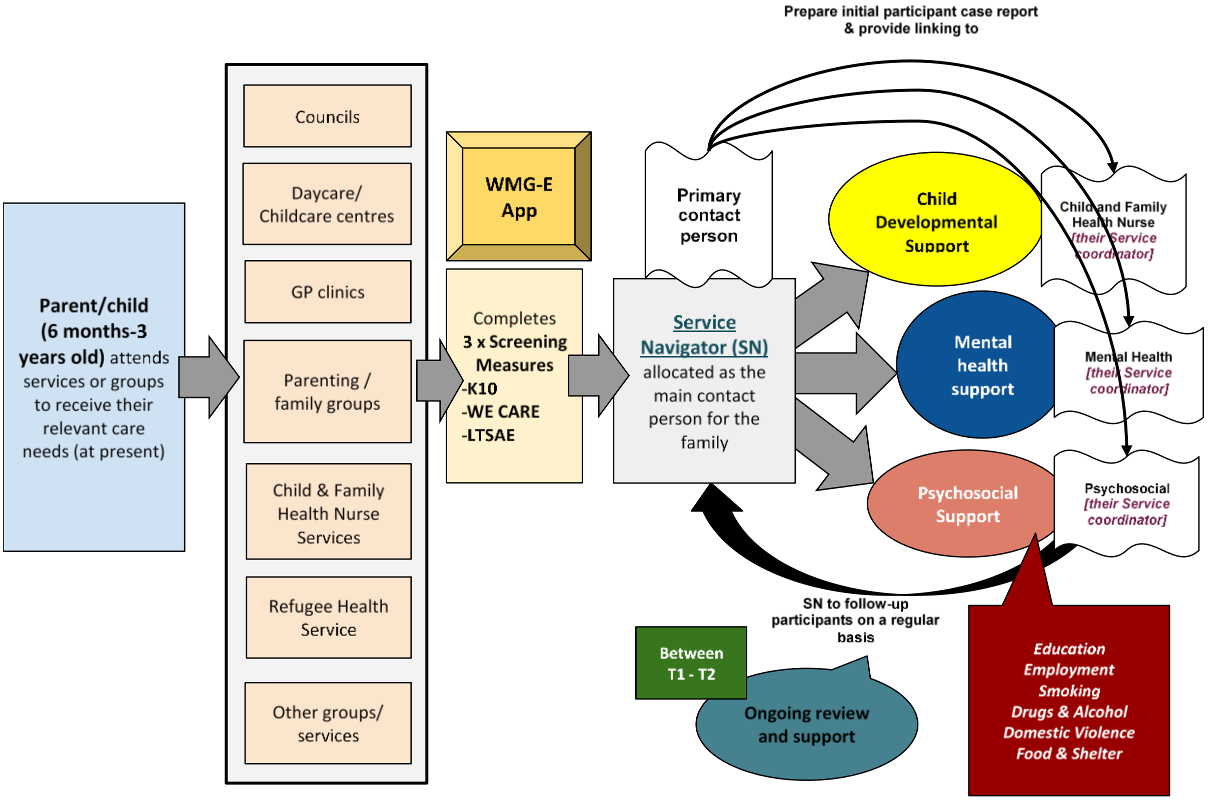

Legends: In the WMG-E program, Time 0 (T0) is at recruitment; Time 1 (T1) is at 6 months' time point; K10 (Kessler-10 measures the current state of parental mental health); WE CARE (to measure the current state of family psychosocial concerns); LTSAE (Learn the Signs Acts Early measures the current state of child development).

Supplementary eFigure 3. Type of unmet needs at baseline by site

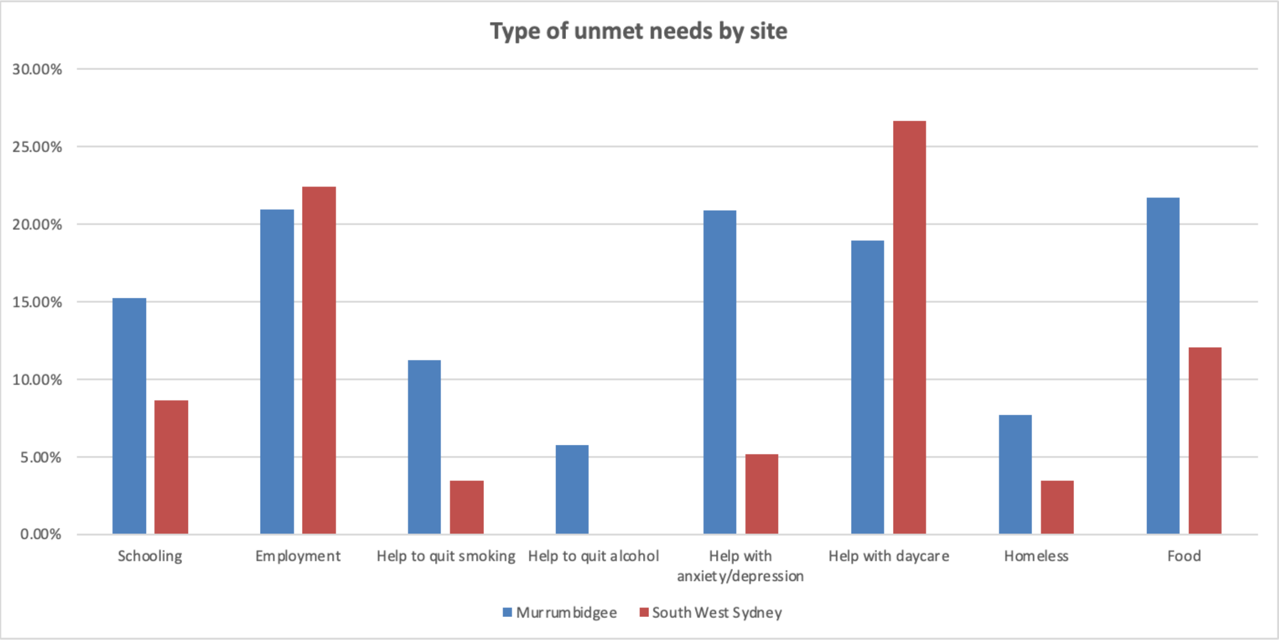

Supplementary eFigure 4. Changes in WECARE scores by treatment and control group

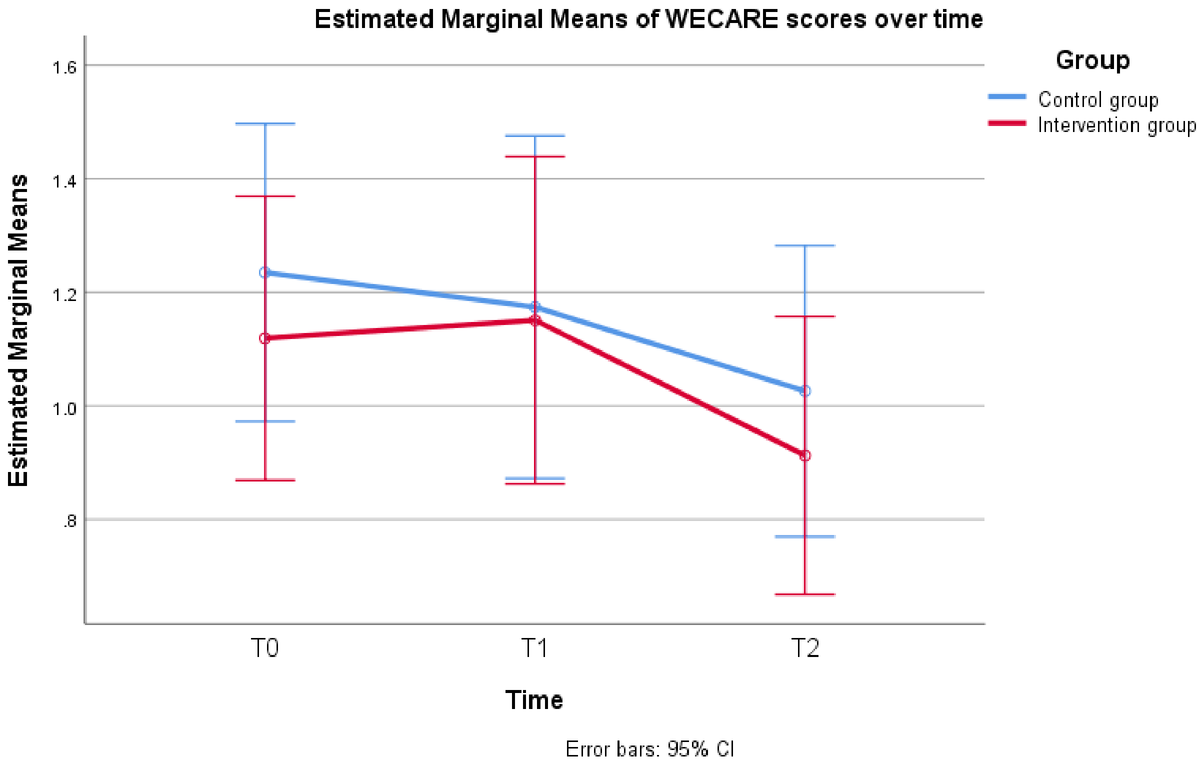

Supplementary eTable 3. Pairwise comparisons of WECARE scores across different timepoints and group

**Contrast**	**Estimate**	**SE**	**p-value**
T0– T1	-0.01	0.07	0.999
T0 – T2	0.19	0.08	**0.042**
T1 – T2	0.19	0.08	**0.041**
Control – Intervention	0.29	0.14	**0.044**

T0 – Baseline, T1 - six-months post-intervention, T2 - 12-months post-intervention

Supplementary eTable 3. WE CARE scores across different timepoints by parental mental wellbeing

Parental mental wellbeing (K10 scores)	Intervention Mean (SD)	Control Mean (SD)	p-value
**Low**	0.74 (1.12)	0.68 (0.75)	0.556
**Moderate**	0.76 (1.61)	0.79 (0.92)	0.840
**High/very high**	2.03 (1.99)	2.46 (1.88)	0.056
